# A Pulley-Assisted Ligature Technique for Safe and Versatile Vascular Division During Uniportal Video-Assisted Thoracic Surgery

**DOI:** 10.1093/icvts/ivag063

**Published:** 2026-03-01

**Authors:** Taichiro Goto, Yuri Natori, Shunsuke Shimizu, Rumi Higuchi, Takahiro Nakagomi

**Affiliations:** Lung Cancer and Respiratory Disease Center, Yamanashi Prefecture Central Hospital, Yamanashi 400-8506, Japan; Lung Cancer and Respiratory Disease Center, Yamanashi Prefecture Central Hospital, Yamanashi 400-8506, Japan; Lung Cancer and Respiratory Disease Center, Yamanashi Prefecture Central Hospital, Yamanashi 400-8506, Japan; Lung Cancer and Respiratory Disease Center, Yamanashi Prefecture Central Hospital, Yamanashi 400-8506, Japan; Lung Cancer and Respiratory Disease Center, Yamanashi Prefecture Central Hospital, Yamanashi 400-8506, Japan

**Keywords:** uniportal VATS, pulmonary vessel division, loop ligature, pulley technique, minimally invasive thoracic surgery

## Abstract

Uniportal video-assisted thoracoscopic surgery (uVATS) provides excellent postoperative outcomes but limits instrument triangulation, making vascular stapling particularly challenging. We developed a simple “pulley technique” using a loop ligature device to create gentle, multidirectional, and dynamically adjustable traction during pulmonary vessel division. This technique involves passing a vessel tape around the target vessel through a loop ligature device and externalizing the tape to allow controlled retraction. The system stabilizes the vessel, improves the angle for stapler insertion, and minimizes the risk of overstretching. Compared with conventional traction methods, the pulley mechanism reduces instrument collision and provides more predictable tension distribution. The technique was successfully applied in multiple lobectomies and segmentectomies without vascular injury or conversion. Its simplicity, reproducibility, and low cost make it a valuable adjunct for improving safety and efficiency in uVATS.

## INTRODUCTION

Uniportal video-assisted thoracoscopic surgery (uVATS) has become an established approach for anatomical lung resection because of reduced postoperative pain, faster recovery, and excellent cosmetic outcomes compared with multiportal video-assisted thoracoscopic surgery (VATS).^1–3^ However, the single incision inherently restricts triangulation, forcing the surgeon to work through a narrow coaxial corridor. This limitation is most apparent during pulmonary vessel division, where precise stapler alignment is critical to avoid vessel injury.

Vascular traction using a vessel loop or forceps can be helpful, but in uVATS, the retraction vector often overlaps with the stapler trajectory, leading to instrument collision, suboptimal stapler angle, and excessive force applied to the vessel. These challenges are further amplified in patients with a narrow intercostal space, obesity, or deeply seated artery branches.

To address these limitations, we developed a simple pulley technique using a loop ligature device. By routing the vessel tape through a loop that acts as a low-friction pulley, we create a dynamic traction system capable of fine directional control without excessive tension. This technical note details the operative steps, biomechanical rationale, and comparative advantages over traditional traction techniques (**Video 1**).

## SURGICAL TECHNIQUE

### Preparation and port strategy

This method was approved by our institutional review board (IRB) on July 30, 2024 (Approval No. 2024-clin-07-01), and has been performed after obtaining informed consent from the patients. A single 3-4 cm incision is placed in the fourth or fifth intercostal space along the anterior axillary line. A wound protector is inserted. The camera is positioned posteriorly, while the working instruments and stapler are introduced anteriorly. The operator stands anterior to the patient, and the assistant stands posteriorly, enabling intuitive coordination of traction and visualization.

### Step 1: Vessel encirclement

After completing lymph node dissection and exposing the target vessel—typically a pulmonary artery segmental branch—the vessel is circumferentially encircled with a soft vessel tape. Care is taken to avoid excessive manipulation, especially in fragile arterial branches with thin adventitia.

### Step 2: Pulley setup

A loop ligature device (Endoloop type; absorbable or non-absorbable depending on preference) is introduced through the incision. The vessel tape is threaded through the loop of the ligature device. The external end of the tape is then withdrawn through the incision and held with a small mosquito clamp. This creates a simple yet effective pulley system: the vessel tape becomes the traction element, and the loop ligature functions as a fulcrum with low friction.

### Step 3: Controlled traction

**Figure 1.**
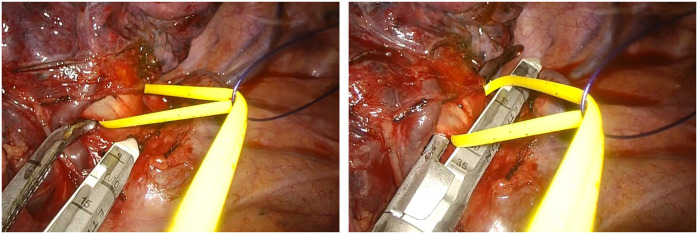
Intraoperative View of the Pulley Setup. The vessel tape passed through the loop ligature device provides gentle, multidirectional, and dynamically adjustable traction to facilitate safe stapler insertion in uVATS. Abbreviation: uVATS, uniportal video-assisted thoracoscopic surgery.

Adjusting the external position of the tape changes the direction and magnitude of traction. Pulling the tape cranially elevates posterior arterial branches; pulling caudally improves exposure for anterior branches. Subtle anterior or posterior pulls fine-tune the angle for stapler insertion. Unlike a simple vessel loop, which produces a fixed traction vector, the pulley allows real-time micro-adjustments without inserting additional instruments (**Figure 1**).

### Step 4: Vascular division

Once an optimal view is obtained, the stapler is introduced through the uniportal incision. The pulley traction opens a smooth, unobstructed passage behind the vessel. After confirming safe positioning, the external tape is released and removed before firing the stapler. The vessel is then safely divided.

## RESULTS

The pulley traction system has been applied in 65 consecutive uVATS procedures including lobectomy and segmentectomy (**Video 2**). In total, this pulley-assisted technique has been used 133 times. In all cases, stable exposure was achieved, and appropriate stapler alignment was easily maintained. No vascular injuries, stapler misfires, or conversions to multiportal or open surgery occurred.

Surgeons reported improved visualization particularly in:

- deep segmental branches such as A6 or A10
- Pulmonary artery branches crossing behind the bronchus
- obese patients with thick chest walls
- narrow intercostal spaces where instrument crowding is pronounced

Operative time was comparable to standard uVATS cases. No additional port or special equipment was required.

## DISCUSSION

### Technical advantages over traditional traction

Conventional vessel-loop traction in uVATS is limited by:

1. Fixed traction vector: that cannot be easily modified
2. Instrument crowding: as the assistant’s traction tool occupies the uniportal corridor
3. High tension: sometimes required to expose the vessel, risking adventitial tearing
4. Stapler obstruction: because the loop often overlaps the stapler path

The pulley technique alleviates all of these limitations.

The loop ligature acts as a mechanical pulley, distributing tension evenly and reducing the required force. Because the traction is applied externally, no extra instrument occupies the port, avoiding instrument collision. Furthermore, the retraction vector can be modified dynamically by simply changing the position of the pulley.

### Comparison with multiportal and robotic approaches

In multiportal VATS, triangulation allows a dedicated instrument to retract the vessel away from the stapler. In robotic surgery, articulating instruments provide even greater flexibility. However, these advantages require additional ports or robotic platforms.

The pulley technique provides comparable stability and directional control without the cost or complexity of additional instrument arms. It essentially mimics a multiportal “third hand” by externalizing the traction vector.^4^^,^^5^

### Biomechanical rationale

The pulley reduces friction and allows controlled redirection of force. When the tape passes through the loop ligature, the traction is divided into 2 components:

- Longitudinal tension: stabilizing the vessel
- Lateral redirection: improving exposure

The friction coefficient of the loop ligature is low, enabling smooth movement with minimal force. This reduces the risk of overstretching or avulsion.

Additionally, the dynamic adjustment allows the operator to modulate tension in response to subtle changes in vessel mobility. This fine control is particularly valuable around small-caliber PA branches where excessive traction is dangerous.

### Educational value

For surgeons early in their uVATS learning curve, the stapling phase is often the most stressful component. The pulley system stabilizes the operative field and enhances predictability, making the learning experience safer and more controlled.^6^ Because the technique uses only standard equipment, it can be readily adopted in any center.

## CONCLUSION

The pulley technique using a loop ligature device provides safe, flexible, and reproducible traction for pulmonary vessel division in uniportal VATS. Its biomechanical advantages, cost-effectiveness, and ease of use make it an excellent adjunct to enhance exposure, reduce instrument collision, and facilitate precise stapler application. This method represents a valuable addition to the armamentarium of minimally invasive thoracic surgery and may help shorten the learning curve for uVATS vascular handling.

## Data Availability

The data underlying this article cannot be shared publicly for the privacy of individuals that participated in the study. The data will be shared on reasonable request to the corresponding author.
